# Severe wildfire exposes remnant peat carbon stocks to increased post-fire drying

**DOI:** 10.1038/s41598-019-40033-7

**Published:** 2019-03-06

**Authors:** N. Kettridge, M. C. Lukenbach, K. J. Hokanson, K. J. Devito, R. M. Petrone, C. A. Mendoza, J. M. Waddington

**Affiliations:** 1 School of Geography, Earth and Environmental Sciences, University of Birmingham, Edgbaston, Birmingham B15 2TT UK; 2 Department of Biological Sciences, University of Alberta, Edmonton, AB T6G 2E9 Canada; 3 School of Geography and Earth Sciences, McMaster University, Hamilton, ON L8S 4K1 Canada; 4 Department of Earth and Atmospheric Science, University of Alberta, Edmonton, AB T6G 2E3 Canada; 5 Department of Geography and Environmental Management, University of Waterloo, Waterloo, ON N2L 3C5 Canada

## Abstract

The potential of high severity wildfires to increase global terrestrial carbon emissions and exacerbate future climatic warming is of international concern. Nowhere is this more prevalent than within high latitude regions where peatlands have, over millennia, accumulated legacy carbon stocks comparable to all human CO_2_ emissions since the beginning of the industrial revolution. Drying increases rates of peat decomposition and associated atmospheric and aquatic carbon emissions. The degree to which severe wildfires enhance drying under future climates and induce instability in peatland ecological communities and carbon stocks is unknown. Here we show that high burn severities increased post-fire evapotranspiration by 410% within a feather moss peatland by burning through the protective capping layer that restricts evaporative drying in response to low severity burns. High burn severities projected under future climates will therefore leave peatlands that dominate dry sub-humid regions across the boreal, on the edge of their climatic envelopes, more vulnerable to intense post-fire drying, inducing high rates of carbon loss to the atmosphere that amplify the direct combustion emissions.

## Introduction

Peatlands have persisted across the globe for millennia, accumulating and storing atmospheric carbon. This persistence has resulted from the ability of these ecosystems to regulate their water content^1^, retaining peat under saturated conditions in response to external perturbations and preventing the propagation of system instabilities that could otherwise have resulted in the ecological collapse, and release of globally important carbon stocks^2,3^. Stabilising feedbacks that regulate peatland water contents have therefore been imperative to peatland persistence^4^. However, global climatic and environmental conditions will test the limits of these feedback responses, as peatlands are pushed outside of their current climatic envelopes. Enhanced high-latitude warming will increase rates of potential evapotranspiration (PET). If unrestricted by internal feedbacks^5^, this will induce peatland drying^6^ and initiate the growth of productive forests that may further intensify water loss^7^. An increased forest canopy (fuel load) combined with reduced peat moisture contents will also increase peatland wildfire severities^8^. This forms peat profiles that are more sensitive to drying^9^ and so further exacerbating the climate driven impacts. With such potential vulnerabilities, there is an immediate need to stress-test^10^ the core feedback mechanisms within peatlands to ascertain their capability to maintain their regulating function under future extreme conditions. Peatland moss evaporation represents one such critical feedback.

The water content of peatlands at the edge of their climatic envelope across the dry sub-humid climatic regions of the circumpolar boreal is often controlled by a covering of feather moss. Feather moss restricts the transport of water to the peatland surface, limiting evaporation and maintaining saturated conditions at depth^11^. In comparison, *Sphagnum* mosses provide an enhanced connectivity with the saturated zones and are associated with higher rates of evaporation^11^. Post-fire, the restriction in feather moss evaporation is reinforced^12^, limiting drying and supporting saturated conditions when these ecosystems are most vulnerable to ecological shifts^2^. However, the extent to which this important feedback holds under future extremes is uncertain, most notably, how the hydrological functioning of near-surface moss layers may be altered in response to projected increases in burn severity. Severe wildfires may burn through the protective moss layer and leave peatlands unprotected to high rates of potential evaporation.

To test the future persistence of the evaporative feedback and determine whether post-fire evapotranspiration (ET) is dependent on burn severity (depth of burn), we measured post-fire ET hourly over the entire growing season across a peatland burn severity gradient within Alberta’s Boreal Plains one year after fire. Burn severity varies widely between the interior and margins of peatlands, with depth of burns ranging from 0.0 to 0.75 m^13,14^. We utilize this fine scale variability in the depth of burn, and measured post-fire ET in three plots within four separate zones of burn severity class within a given peatland (all areas within the study area burned but to varying degrees allowing comparison). Measurements were conducted in three areas of assumed pre-fire feather moss peat: i) *low burn severity* plots with a burn depth less than 0.05 m and residual feather moss visible; ii) *moderate burn severity* where the depth of burn was greater than 0.05 m, consistent with burns projected under future climates^8^; and iii) *high burn severity* in which the peat had been burned down to underlying mineral soil, with burn depths up to 1.0 m^13^. For comparison, measurements were also conducted within a zone of *Sphagnum* moss peat, burned at a low severity, that more weakly restricts the supply of water to the evaporating surface^12^.

To identify the potential for severe burns projected under future climates to substantially increase drying, we simulated post-fire peatland-scale ET under varying burn severities (average burn ranging from zero to 0.3 m in depth). The model assumes a 0.15 m deep feather moss layer overlying a *Sphagnum* peat profile. Post-fire ET is calculated based upon: i) the average daily ET of the remnant burned surface cover (assumed equal to low burn severity feather moss if part of the pre-fire feather moss layer is retained or moderate burn severity peat if the feather moss layer is entirely combusted), and ii) the proportion of the post-fire peatland surface composed of these different peatland units under varying burn severity distributions.

## Results

**Figure 1:**
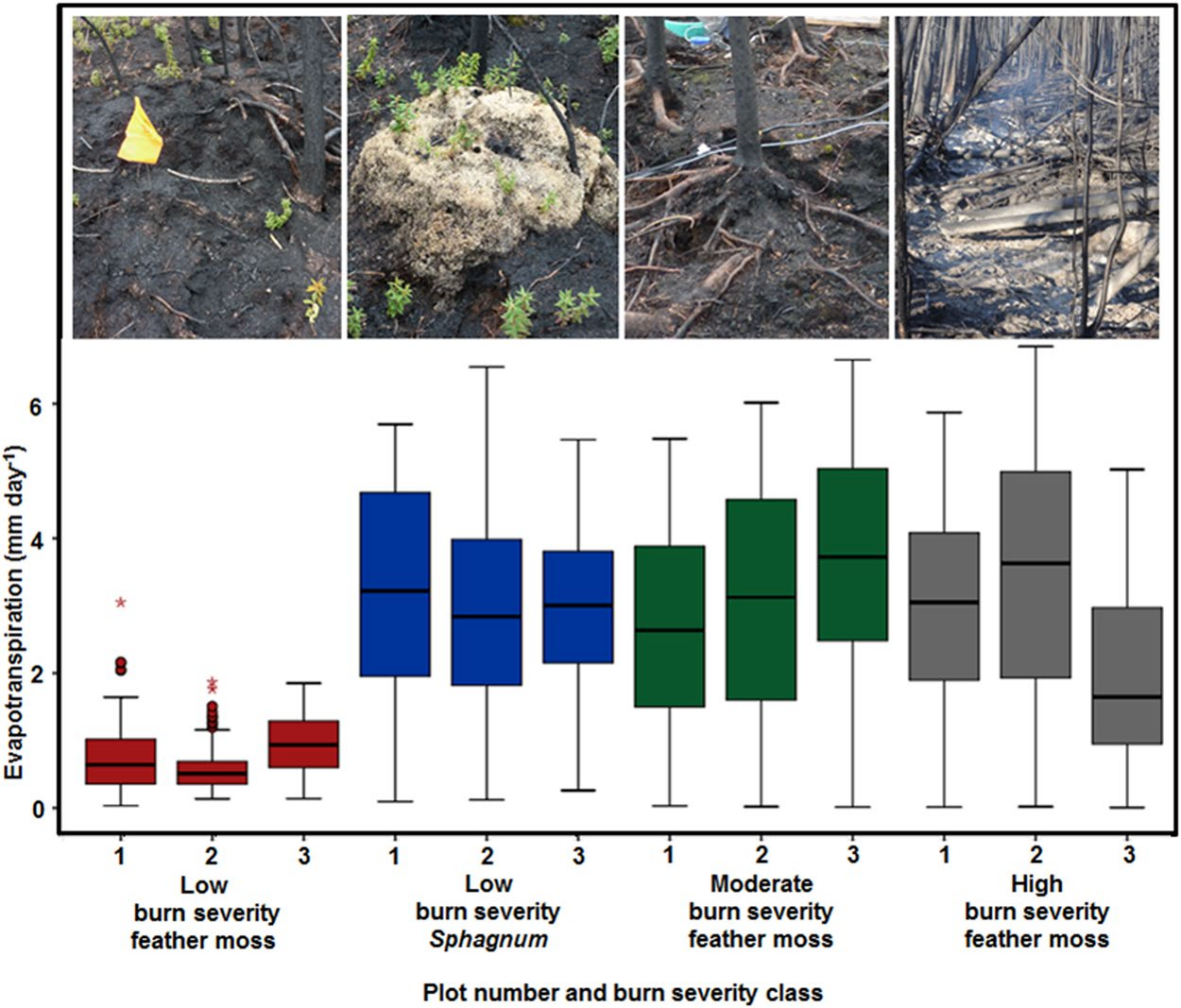
Daily evapotranspiration within each of the three plots for: (i) low burn severity feather moss, (ii) low burn severity *Sphagnum*, (iii) moderate burn severity feather moss and (iv) high burn severity feather moss zones over the growing season one year after fire. Pictures provide graphical representation of the four zones.

ET was 410% higher in the moderate burn severity (ET = 3.12 ± 0.38 mm d^−1^; t = 6.14, p < 0.001) and 363% higher in the high burn severity plots (ET = 2.76 ± 0.38 mm d^−1^ t = 5.19, p < 0.001) than the low burn severity feather moss plots (ET = 0.76 ± 0.27 mm d^−1^) (Fig. 1). In accordance with^12^, ET was significantly higher in the low burn severity *Sphagnum* plots than the low burn severity feather moss plots (p < 0.001; t = −5.91; Fig. 1). ET averaged 0.76 ± 0.27 mm day^−1^ within the feather moss plots, compared with 3.03 ± 0.38 mm d^−1^ within *Sphagnum*. There was no significant difference in daily ET between the low severity *Sphagnum* plots and either the moderate burn severity (ET = 3.12 ± 0.38 mm d^−1^, t = 0.22, p = 0.82) or high burn severity plots (ET = 2.76 ± 0.38 mm d^−1^, t = −0.711, p = 0.50).

**Figure 2:**
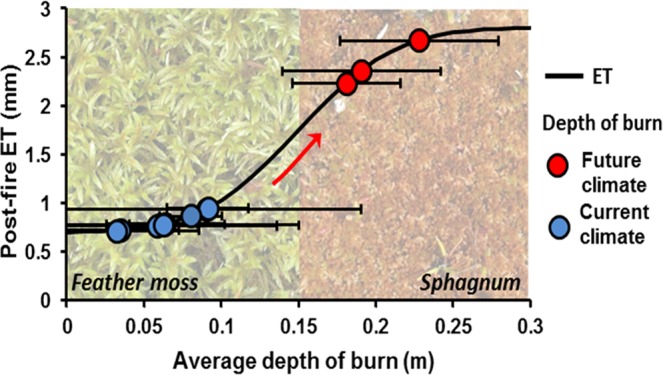
Simulated peatland evapotranspiration (ET) for burn depths ranging between 0 and 0.3 m (black solid line). Pre-fire feather moss – *Sphagnum* transition within the simulated peatland at a depth of 0.15 m (as pictured). Measured burn depths for peatland interiors observed across Alberta, Canada (blue circles; mean ± standard deviation^8,13–16^ with associated simulated post-fire ET. Future climate (red circles) represent burn depths observed by^8^ within a moderately drained peatland indicative of peatland ecology, hydrology and fire severities projected under future climates. Simulated ET does not represent a prediction for individual sites which represent a broad range in hydrological conditions and feather moss surface covers.

Simulated post fire surface cover ranged from 100% feather moss to 100% exposed *Sphagnum* peat over the range of prescribed burn severities (Fig. 2; solid line). The resultant relationship between ET and burn severity is strongly nonlinear, with a break point in post-fire ET simulated at an average burn depth of 0.10 m. Above this break point, post-fire ET markedly increases with burn depth. Within peatland interiors, current burn depths^8,13–16^ across northern Alberta fall below the threshold (blue circles; Fig. 2). However, burn severity is higher in plots burned after a decade of drying, indicative of future climatic conditions (Fig. 2, red circles^8^). Burn severities representative of future climates exceeds the ET threshold within a feather moss peatland (Fig. 2).

## Discussion

Moderate and high severity burning overrides the important stability mechanism of reduced post-fire evaporation that protects feather moss dominated peatlands typical of southern continental boreal regions from drying^12^. While PET is high following wildfire due to the open forest canopy^17^, actual water loss to the atmosphere is greatly restricted under low severity burns within feather moss peat profiles^12^. When burn severity is moderate or high, we show that the stabilising response is exceeded and the peatland evaporates relatively freely, equivalent to an open *Sphagnum* surface.

**Figure 3:**
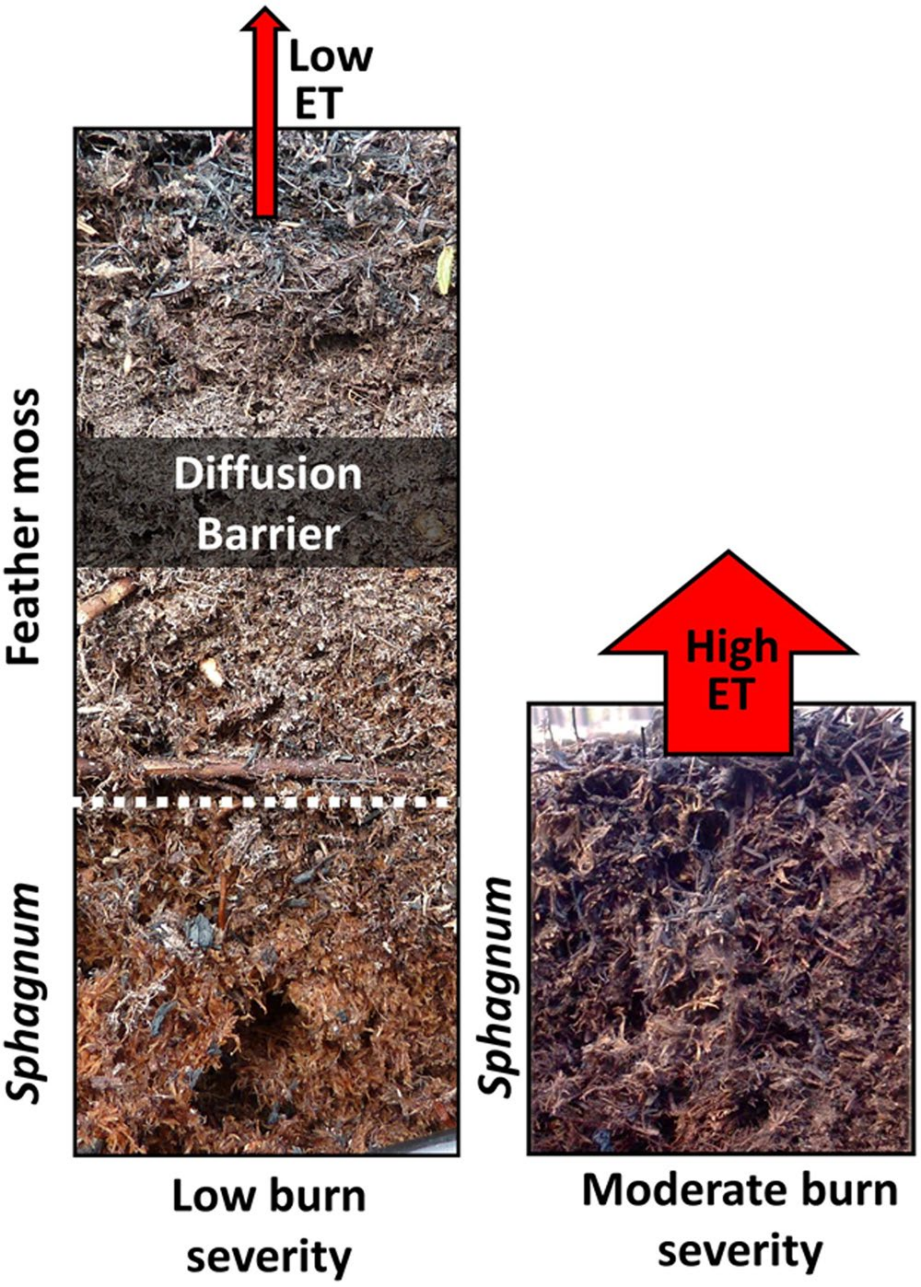
Conceptualisation of peat profile in response to fire. Left, low burn severity that leaves the feather moss profile intact, acting as a diffusion barrier through which water from the wet peat beneath must travel, limiting evapotranspiration (ET). Right, moderate burn severity that has removed feather moss peat through combustion exposing the *Sphagnum* moss beneath. The profile is able to evaporate relatively freely, comparable to a singed *Sphagnum* profile.

We hypothesize that the layered structure of the peat profile controls the transition between low and high ET. Boreal peatlands show a typical successional behaviour over a fire interval. *Sphagnum* species increase their surface cover and dominate 20 years after fire^18^. Tree canopy growth subsequently reduces light availability in the sub canopy, driving secondary succession to feather moss 60 to 80 years post fire^18^. The precise percentage cover and timing of this transition depends on tree growth rates, tree densities and the hydrological setting of the peatland^19–21^. However, vegetation succession produces a layered pre-burned stratigraphy, with feather moss overlaying *Sphagnum* peat. A low burn severity is considered to leave the overlying feather moss layer intact to act as a barrier to water transport that restricts post-fire evaporation^12^ (Fig. 3a). When burn depth extends below the feather moss layer it exposes either the *Sphagnum* peat beneath or the mineral soil below. This transition is likely associated with the shift in the peatland to a less restricted, high ET state (Fig. 3b). Within peatland interiors, current burn depths across northern Alberta fall below the threshold. However, burn severity is higher in plots burned after nearly two decades of drying, indicative of future climatic conditions (Fig. 2, red circles^8^). Within a feather moss peatland, this increased burn severity projected under future climates exceeds the ET threshold, increasing simulated post-fire drying by weakening the stabilizing function of the feather moss layer (Fig. 2).

Burned feather moss restricts post-fire evaporation, supports saturated conditions and so protects the peatland carbon stock. However, we found that this regulating function of feather moss could fail with further climate stress. With climate change mediated drying, and the associated increase in burn severity, we argue that these peatlands will likely transition to a more freely evaporating state following wildfire. Under this new state, the post-fire restriction on ET would be reduced during periods of high PET from the peat surface, resulting from the open burned canopy^17^. Increased ET, combined with an increased sensitivity to water loss resulting from the combustion of the porous (high specific yield) near surface moss layer^9^, will drive lower water table positions. This assumes that the hydraulic connection between the saturated peat and the evaporating surface is effectively maintained^5^ and wider ecohydrological feedbacks are not invoked to further restrict water loss^1^. Such drying will expose remnant peat carbon stocks to aerobic conditions, increasing rates of decomposition and further enhance carbon losses associated with the fire. It will also improve the seed bed quality, promoting rapid post-fire growth of deciduous species that may interrupt the fire ecology cycle^22^, supporting dryer conditions by enhancing post-fire transpiration and promoting rapid fuel load accumulation to support a potential transition to a high frequency, low intensity fire regime^2^.

## Methods

### Study site

Measurements were conducted within the Utikuma Lake Region Study Area in north-central Alberta (56.107°N 115.561°W), within a coarse-textured outwash plain^23^. Measurements were undertaken within a small (60 m by 150 m) peatland surrounded by aspen forest^13^. The peatland was burnt in May 2011 in the ~90,000 ha Utikuma Complex forest fire. Depth of burn varied from 0.00 to 1.10 m across the site^13^. Prior to the fire, the burned peatland was dominated by feather moss (*Pleurozium schreberi*) lawns with some *Sphagnum fuscum* hummocks underlying a vascular vegetation cover of *Rhododendron groenlandicum* and *Rubus chamaemorus*. There was a dense black spruce tree canopy of ~7,000 stems per hectare across the peatland. The margin was characterised by a zone of feather moss with a vascular vegetation cover of *Rhododendron groenlandicum* and *Rubus chamaemorus* that may have transitioned to a riparian swamp bordering the forest upland (from inspection of similar unburned sites within the vicinity^26^).

Following fire the site was classified into four zones associated with the pre-fire vegetation cover, distinct visual zones of burn severity and distance from the peatland-upland interface. Feather moss cover plots were discretized into low, moderate and high burn severity zones. Residual feather moss remained visible within low burn severity zones located principally within the middle of the peatland, with a burn depth less than 0.05 m. Moderate burn severity zones were defined as zones where the depth of burn was greater than 0.05 m but in which a peat surface remained. These zones are consistent with an increase in depth of burn projected under future climates^8^. Zones of high burn severity were located at the extreme margin of the peatland and were defined as regions in which the peatland had burned through to the mineral soil beneath.

### Hydrological and micrometeorological measurements

Average post-fire growing season evapotranspiration (ET) was measured within a feather moss dominated peatland under a range of burn severities every hour throughout the 2012 growing season (May to August inclusively), approximately one year following wildfire. Measurements were conducted using an automated version of the chamber approach of  ^24^. Three Perspex chambers, with 0.2 m^2^ surface area, were installed within each designated zone. To measure ET, the chamber was closed for two minutes and the air within the chamber continuously mixed by a fan. ET was calculated from the rate of increase in humidity within the closed chamber of known volume^5^ measured using an infra-red gas analyser (Li-COR LI-840). The control of the different measurement zones (Feather moss; low, moderate and high burn severity: *Sphagnum* low burn severity) on daily ET were analysed using a linear mixed effects model in R (nlme)^27^, with the zone as a fixed effect and chamber as a random effect to account for the lack of independence among measurements.

### Peatland ET modelling

The simulated peatland was 1.0 m deep and composed of a feather moss layer overlying a *Sphagnum* peat profile. Across the peatland the transition from feather moss to *Sphagnum* peat occurred at a depth of 0.15 m. This is equivalent to 50 years of feather moss growth, assuming organic matter storage of 4 kg m^−2^ over 50 years at a bulk density of 27 kg m^−3^^ 25^. The defined peatland was exposed to a range of isolated fires of different severities, with average burn depths ranging from 0.0 to 0.3 m. Within a single fire, the burn depth varied across the peatland. The burn depth was assumed to be normally distributed with a standard deviation of 0.05 m; average standard deviation observed within Albertan peatlands^8,13–16^. This results in post-fire surfaces that, dependent on the average burn depth, varied from 100% singed feather moss to 100% exposed *Sphagnum* peat. *ET* was calculated based on the proportion of the surface composed of *Sphagnum* and feather moss and the associated average ET of each. Thus ET was equal to:

$$ET=E{T}_{LS}{\int }_{0}^{0.15}B(x)dx+E{T}_{SB}{\int }_{0.15}^{1.0}B(x)dx,$$

where *B* is the burn depth distribution across the peatland, *x* the depth, and subscripts *LS* and *SB* indicate average growing season ET for low burn severity and moderate burn severity feather moss peat, respectively.
